# Deep Venous Thrombosis and Pulmonary Thromboembolism Associated With Retroperitoneal Hematoma in a Patient With Ehlers-Danlos Syndrome Type VI

**DOI:** 10.7759/cureus.32750

**Published:** 2022-12-20

**Authors:** Catarina Pereira, Fernando Nogueira, José Cunha Marques, José Pestana Ferreira, Jorge S Almeida

**Affiliations:** 1 Internal Medicine, Centro Hospitalar Universitário de São João, Porto, PRT; 2 Medicine, Faculdade de Medicina da Universidade do Porto (FMUP), Porto, PRT

**Keywords:** thrombosis, anticoagulation, hematoma, pulmonary thromboembolism, ehlers-danlos syndrome

## Abstract

Ehlers-Danlos Syndrome (EDS) is a group of genetic diseases of the connective tissue, which is rare and is characterized by joint hypermobility, tissue, and vascular fragility.

We present the case of a 38-year-old woman with a known diagnosis of EDS type VI who came to the emergency room, complaining of sudden dyspnea in the context of abdominal pain and pain in the left lower limb with one week of evolution. Computed axial tomography showed the presence of bilateral pulmonary thromboembolism, iliofemoral thrombosis, and a retroperitoneal hematoma. Anticoagulation was started and the patient was admitted to the intermediate care ward.

This case highlights the need to know the implications of increased vascular fragility in type VI EDS, with potentially serious consequences. Underlying is the inevitable need for judicious consideration of the decision to undergo anticoagulation, in the context of a retroperitoneal hemorrhagic event and pulmonary thromboembolism.

## Introduction

Ehlers-Danlos Syndrome (EDS) includes a series of rare diseases of genetic etiology, which are characterized by hyper flexibility, joint hypermobility, and tissue and vascular fragility. The accepted classification uses both the mutations identified and the phenotype [1-3]. The prevalence of this syndrome is about 1 in 5000 births [4].

Vascular events are associated with subtypes IV and VI, especially type IV-vascular EDS. Without a cure, the therapeutic approach is mainly based on surveillance for early diagnosis of complications resulting from the disease (vascular, ophthalmological, musculoskeletal). Type I is a classical EDS with severe skin involvement, an autosomal dominant disorder with a defect in type V collagen. In type III the patients have a defect in tenascin X, with severe joint hypermobility problems. In type IV (classically vascular) the patients present major arteries involvement and cutaneous and articular involvement because of the defect in collagen III. Ocular-scoliotic EDS (type VI) is characterized by the involvement of a defect in the lysyl hydroxylase relative to prolyl hydroxylase activity and the patients affected present musculoskeletal changes and greater likelihood of rupture of the eyeball and large vessels and may also have a presentation similar to types I and II. All types of EDS in genetic terms are autosomal dominant, with the exception of type VI whose inheritance is autosomal recessive and another type linked to the X whose pathogenic mechanisms, namely the protein involved are not well known [3-6].

The prognosis depends on organ involvement, with a lower average life expectancy in subtypes IV and VI, precisely because of the potential for vascular involvement, which may result in relevant morbidity and mortality, often at a young age (<40 years old) [6].

## Case presentation

We present the case of a 38 years old asymptomatic woman, with homozygosity for the procollagen-lysine, 2-oxoglutarate 5-dioxygenase 1 (PLOD1) gene mutation. She is the daughter of consanguineous parents and an older sister who died at 38 years old in the context of spontaneous rupture of an abdominal aortic aneurysm, which motivated the family genetic study. On physical examination, the patient only presented cutaneous hyperdistensibility and joint hypermobility. So the diagnosis of EDS type VI was made, according to the Villefranche classification [1,2].

The patient presented to the emergency department due to sudden onset dyspnea. On questioning, she reported one week of abdominal pain in the left iliac fossa and the left lower limb with edema. She did not have a history of trauma.

**Figure 1:**
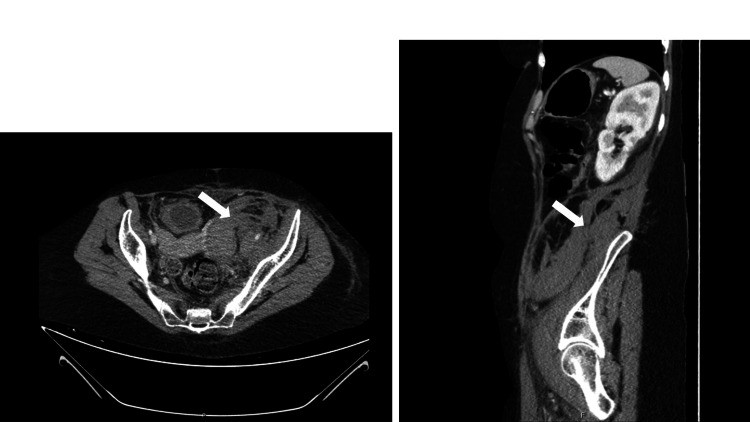
Admission axial computerized tomography (axial and sagittal planes). Arrows indicate the hematoma

Laboratory studies showed acute anemia (hemoglobin 8.6 g/dL for a previous value of 14.8 g/dL (six months ago), increased inflammatory parameters (leukocytosis 17.16x10^9/L (normal range 4.0-11.0x10^9), C-reactive protein 131.3 mg/L (normal value <3 mg/L)), increased D-dimer (4.4 mcg/mL, normal value <0.5 mcg/mL), without elevation of cardiac biomarkers or B-natriuretic peptide and normal arterial blood gas. Doppler ultrasound of the left lower limb revealed a long left femoral deep vein thrombosis (involving the internal and external iliac veins to the left common iliac vein). Computerized tomography (CT) axial angiography confirmed these findings and demonstrated as well filling defects of the pulmonary arteries, involving the basal segmental branches of the right lower lobe, an anterior segmental branch of the left upper lobe, and a proximal segment of the basal segmental branches of the left lower lobe, suggesting bilateral segmental thromboembolism. The CT angiography also documented a hematoma centered in the left inguinal region and left iliac fossa and extending superiorly to the inferior pole of the left kidney, with 97x67x170 mm^3^ (TxAPxL) (Figure 1).

Transthoracic echocardiogram revealed slight dilatation of the right chambers, with preserved biventricular function and no other abnormalities, namely no intracavitary thrombi.

The patient was started anticoagulation with unfractionated heparin and was admitted to an intermediate care unit. On admission to this unit, the hemoglobin was Hb 7.0 g/dL, and was given one unit of red blood cells with good transfusion recovery; there was no further need for additional transfusion support. All additional studies, including the autoimmune screen (including celiac disease and inflammatory bowel disease), prothrombotic study, or nutritional deficiencies were negative.

**Figure 2:**
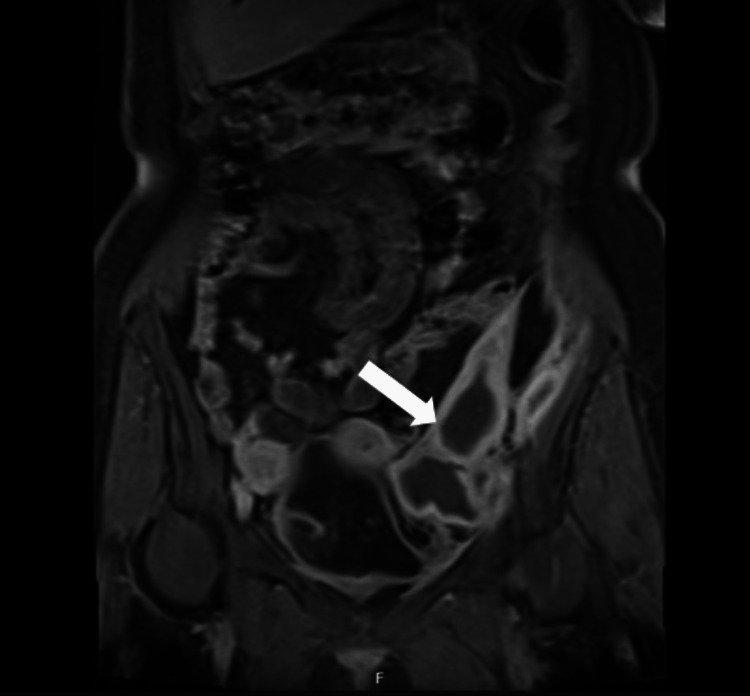
Magnetic resonance imaging - Fat-suppressed contrast-enhanced T1-weighted image in the coronal plane showing a multiloculated hematoma along the left iliac muscle and in the left iliac fossa. Arrow indicates the hematoma

She was transferred to the Internal Medicine ward on the sixth day of hospitalization. Reevaluation abdominal CT scans were performed on the fourth and tenth day of hospitalization revealing a slight retroperitoneal hematoma reduction, without any signs of active bleeding. Unfractionated heparin was safely switched to low-weight molecular heparin and to warfarin was performed on the 13th day of hospitalization, without associated bleeding complications. On the 20th day of hospitalization, abdominopelvic magnetic resonance imaging (MRI) was performed, which showed a progressive reduction in the dimensions of the blood collection (72x43x111 mm^3^) and excluded trauma as a probable associated etiological cause, without other alterations (Figure 2).

The MRI still showed extensive thrombosis of the iliac (common, internal, and external) and left femoral veins, stable in comparison with the previous exams. She was discharged on the 24th day of hospitalization, with follow-up by Internal Medicine and Physical Medicine and Rehabilitation, anticoagulated with warfarin.

## Discussion

There are many risk factors for venous thromboembolism, namely immobilization, hospitalization, trauma, and prothrombotic states like autoimmune and inflammatory diseases like inflammatory bowel disease, infection, obesity, etc. [7]. After excluding the most frequent risk factors through an exhaustive study, it was possible to infer that the event was most likely due to the hemorrhagic event, which, when performing compression at the venous level, provided phenomena of stasis leading to a thrombotic event.

Patients with type VI EDS have a high risk of a catastrophic vascular event, which usually occurs before the end of the fifth decade of life. In our patient, the spontaneous (given the absence of a history of major or minor trauma) retroperitoneal hematoma resulted in external vascular compression and subsequent extensive thrombosis iliofemoral, which culminated in pulmonary thromboembolism. Due to the high bleeding risk, therapeutic decisions were made considering quick anticoagulation reversal potential (drug choice, via administration, risk-benefit weighting, timing of introduction, duration of introduction, and monitoring method). So, the selection of hypocoagulant drugs for this patient (unfractionated heparin and warfarin) was essentially due to the possibility of a reversal in case of active hemorrhage, weighing the risk of variability in levels. Despite the high bleeding risk provided by the retroperitoneal hematoma, it was decided to promptly antiocoagulate the patient, considering there was no active bleeding on the CT scan and the patient was hemodynamically stable. This is a rare case, particularly with regard to the therapeutic decision, as there are no similar cases with the same degree of complexity described in the literature. The Pulmonary Embolism Severity Index (PESI) score reveals a very low risk, with 30-day mortality equal to or less than 1.6%. Subsequently, about six months, the patient fell at home, with eye trauma causing unilateral amaurosis and decreased acuity of the contralateral eye. Given the high risk of falls afterward, it was decided, in a decision shared with the patient, to suspend hypocoagulation after six months.

## Conclusions

The clinical practice, and particularly Internal Medicine, involves complex decision-making, many of which result from the sharing of knowledge within each assistant medical team and often by multidisciplinary teams, individualized decisions adapted to the patient involved in the same. This case highlights those decisions that were always debatable, a little linear, and the need for weighing and decision-making the risk-benefit inherent to the medical decision which is always in discussion with a patient who is fully aware of the diagnosis and the prognosis.
